# A New Phenolic Glycoside from the Barks of *Cinnamomum cassia*

**DOI:** 10.3390/molecules191117727

**Published:** 2014-10-31

**Authors:** Junfen Zeng, Yongbo Xue, Yongji Lai, Guangmin Yao, Zengwei Luo, Yonghui Zhang, Jinwen Zhang

**Affiliations:** 1 Tongji Hospital Affiliated to Tongji Medical College, Huazhong University of Science and Technology, Wuhan 430030, China; 2 Department of Pharmacy, Renmin Hospital of Wuhan University, Wuhan 430060, China; 3 Hubei Key Laboratory of Natural Medicinal Chemistry and Resource Evaluation, School of Pharmacy, Tongji Medical College, Huazhong University of Science and Technology, Wuhan 430030, China

**Keywords:** *Cinnamomum cassia*, phenolic glycoside, immunosuppressive activities

## Abstract

A new phenolic glycoside (**1**), named methyl 2-phenylpropanoate-2-*O*-*β*-d-apiofuranosyl-(1→6)-*O*-*β*-d–glucopyranoside, was isolated from the barks of *Cinnamomum cassia*, along with three known phenolic glycosides and four known lignan glycosides. The structure of **1** was elucidated by extensive interpretation of spectroscopic data and chemical method. Selected compounds were evaluated for their immunosuppressive activities against murine lymphocytes. Compounds **1**, **2**, **6** and **8** exhibited differential inhibition against ConA-induced T cells proliferation.

## 1. Introduction

The plant *Cinnamomum cassia* (Lauraceae), which originates in the south of China, is widely cultivated in tropical or subtropical areas, such as Fujian, Guangdong, Guangxi, Yunnan and Hainan Provinces of China, as well as Taiwan, India, Indonesia, Laos, Malysia, Thailand and Vietnam [1]. The bark of *C. cassia*, well known as *Cinnamomi cortex* in China, has long been used as spice and flavoring agents [2]. It also possesses various biological activities, such as insecticidal, anti-allergy, antipyretic, anticancer, anti-Alzheimer′s disease, antidiabetic [3] and immunosuppressive activity [4]. In our previous study [5], three diterpenoids, cinncassiols F and G, and 16-*O*-*β*-d-glucopyranosyl-19-deoxycinncassiol G, with unprecedented carbon skeletons, were isolated from the barks of *C. cassia*. It is notable that two of them showed significant inhibitory effects on the proliferation of murine T cells induced by ConA. Recently, with the aim of discovering more bioactive compounds from *C. cassia*, the extracts of EtOAc and *n*-BuOH were further phytochemically studied, which led to the isolation of a new phenolic glycoside (**1**), together with three known phenolic glycosides (**2**–**4**) and four known lignan glycosides (**5**–**8**). The immunomodulatory activities against murine lymphocytes of the selected compounds were reported in this work.

## 2. Results and Discussion

**Figure 1:**
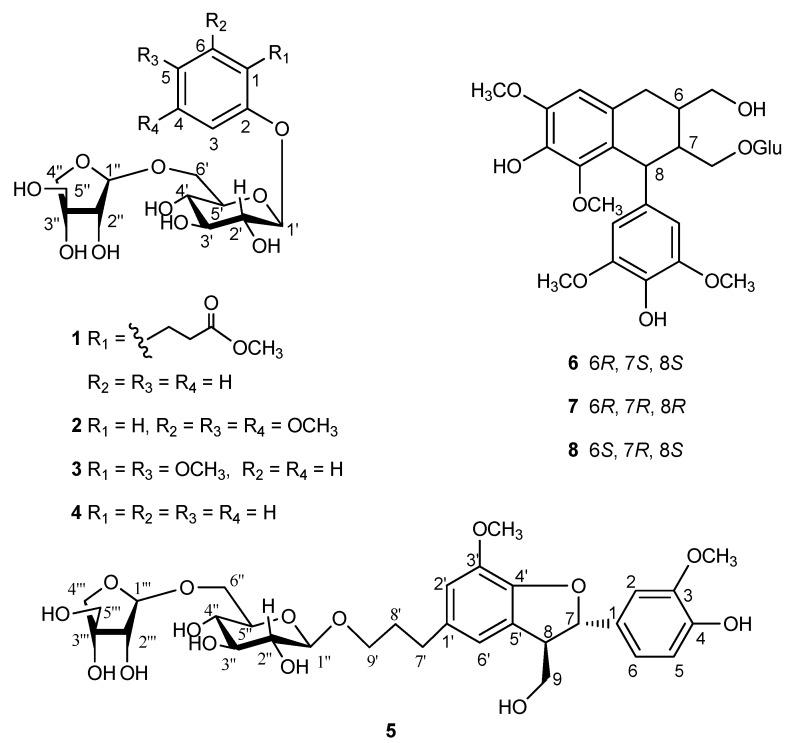
Structures of **1**–**8**.

The EtOAc extract of *C*. *cassia* was separated by various chromatographic techniques to yield a new phenolic glycoside (**1**), along with seven known compounds. The known compounds were identified as 3,4,5-trimethoxyphenol-*β*-d-apiofuranosyl-(1→6)-*O*-*β*-d-glucopyranoside (**2**) [6], cinnacasolide E (**3**) [7], phenol-*β*-d-apiofuranosyl-(1→6)-*O*-*β*-d-glucopyranoside (**4**) [8], samwiside (**5**) [9], (6*R*,7*R*,8*R*)-7a-[(*β*-d-glucopyranosyl)oxy]lyoniresinol (**6**) [10], (6*S*,7*R*,8*R*)-7a-[(*β*-d-glucopryanosyl)oxy]lyoniresinol (**7**) [10], and (6*R*,7*S*,8*S*)-7a-[(*β*-d-glucopyranosyl)oxy]lyoniresinol (**8**) [11], by comparison of their NMR data with those reported in literatures (Figure 1).

Compound **1** was isolated as amorphous powder, with ${\left[\alpha \right]}_{D}^{25}$ −65.1 (*c* = 0.47, MeOH). Its molecular formula, C_21_H_30_O_12_, was deduced from the HRESIMS peak at *m/z* 497.1616 [M+Na]^+^ (calcd for C_21_H_30_O_12_Na, 497.1629). The IR spectrum showed the presence of carbonyl (1719 cm^−1^) and hydroxyl group (3391 cm^−1^). The ^1^H NMR spectrum of **1** (Table 1) showed proton signals for a 1,2-disubstituted aromatic ring (δ_H_ 7.21, dd, *J* = 8.1, 1.2 Hz, H-3; 7.18, ddd, *J* = 8.1, 7.9, 1.9 Hz, H-4; 6.96, ddd, *J* = 7.9, 7.9, 1.2 Hz, H-5; and 7.17, dd, *J* = 7.9, 1.9 Hz, H-6), a methoxyl (δ_H_ 3.66, s). ^13^C-NMR and DEPT spectra (Table 1) of **1** showed 21 carbon signals, including one methoxyl, one ester carbonyl, five methylenes, eleven methines, and three quaternary carbons. Comparison of the ^1^H- and ^13^C-NMR spectra with those of **4** revealed that **1** differed from **4** by the presence of a methyl propionate at C-1. Thus, compound **1** was determined to be an analogue of **4**. The HBMC and ^1^H-^1^H COSY spectra of **1** indicated the presence of a methyl propionate moiety, which was connected at C-1.

The HBMC correlations from H-1′ to C-2 and from H-1′′ to C-6′ revealed the linkages of the two sugars to be the same as those of **4**. The sugars were finally confirmed by comparing the retention times of the three trimethylsilylthiazolidine derivatives obtained from GC analysis. On the basis of the above evidences, compound **1** was determined and named methyl 2-phenylpropanoate-2-*O*-*β*-d-apiofuranosyl-(1→6)-*O*-*β*-d-glucopyranoside.

**Table 1:** ^1^H-(400 MHz) and ^13^C-NMR (100 MHz) Data of **1** in CD_3_OD.

Position	δ_H_	δ_C_
1		131.3
2		157.0
3	7.21 dd (8.1, 1.2)	128.9
4	7.18 ddd (8.1, 7.9, 1.9)	116.7
5	6.96 ddd (7.9, 7.9, 1.2)	123.5
6	7.17 dd (7.9, 1.9)	131.0
7	3.00 m	26.9
8	2.68 t (7.7)	35.3
9		175.9
OMe	3.66 s	52.0
1′	4.88 d (7.8)	102.8
2′	3.50 m	75.0
3′	3.49 m	78.2
4′	3.38 m	71.6
5′	3.60 m	77.0
6′a	4.04 d (10.6)	68.8
6′b	3.64 m	
1′′	5.00 d (2.3)	111.0
2′′	3.93 d (2.3)	78.1
3′′		80.0
4′′a	3.77 d (9.7)	75.0
4′′b	3.97 d (9.7)	
5′′	3.61 s	65.6

The crude extract of *C. cassia* has been reported to exhibit potent anti-complement [12] and immunosuppressive activities [4]. Thus, compounds **1**, **2**, and **5**–**8** were evaluated for their immunomodulatory activities against murine lymphocytes. The results (Table 2) showed that compounds **1**, **2**, **6**, and **8** inhibited the proliferation of ConA-induced murine T cells at a concentration of 50 μM, while the positive control at the concentration of 50 μM, resulted in an inhibitory ratio of 106.1% against T cells and an inhibitory ratio of 75.8% against B cells.

**Table 2:** Effects on Murine Lymphocyte Proliferation Induced by Concanavalin A (ConA) (5 μg/mL) or Lipopolysaccharide (LPS) (10 μg/mL) of the Selected Compounds **1**, **2**, and **5**–**8 ***^a^*.

Compounds	Concentration (μM)	Inhibitory/Enhanced Rate (%) *^b^*	
		ConA−Induced T Cell Proliferation	LPS−Induced B Cell Proliferation
**1**	200	−56.7	10.3
	100	−61.2	c25.2
	50	−35.4	25.6
	25	−22.1	30.1
	12.5	15.2	7.8
**2**	200	−45.2	−35.1
	100	−38.8	−5.8
	50	−8.5	−12.5
	25	−12.5	10.3
	12.5	−20.1	6.2
**5**	200	−27.9	28.4
	100	−5.4	22.2
	50	6.5	10.7
	25	11.7	−1.5
	12.5	2.8	13.8
**6**	200	−47.7	−13.2
	100	−58.0	20.5
	50	−23.6	41.4
	25	−47.5	36.1
	12.5	−11.3	−12.5
**7**	200	−10.4	−22.7
	100	−13.8	78.5
	50	2.5	62.4
	25	18.7	58.4
	12.5	35.1	12.5
**8**	200	−80.1 *	−46.7
	100	−58.3	−10.3
	50	−19.5	25.6
	25	27.8	57.8
	12.5	14.5	52.2
**CsA**	200	−109.8 **	−106.1 **
	100	−106.0 **	−94.8 **
	50	−106.1 **	−75.8 *
	25	−97.1 **	−60.1
	12.5	−84.5 **	−54.5

*^a^* Results are represented as mean ± SD based on three independent experiments. *^b^* -Inhibitory effect; +stimulatory effect. (*n* = 3; * *p* < 0.05; ** *p* < 0.01 compared with control). Positive control: ConA or LPS; negative control: DMSO.

## 3. Experimental

### 3.1. General Procedures

NMR spectra were recorded on a Bruker AM 400 spectrometer (Bruker, Ettlingcn, Germany), and the ^1^H- and ^13^C-NMR chemical shifts were referenced to the solvent peaks for CD_3_OD at δ_H_ 3.31 and δ_C_ 49.15. HRESI-MS data were measured on a LC-LTQ-Orbitrap XL spectrometer (Thermo Fisher, Waltham, MA, USA). Optical rotations were determined on a Perkin-Elmer PE-341LC polarimeter (PerkinElmer, Waltham, MA, USA). IR spectra were recorded on a Bruker VERTEX 70 spectrometer (Bruker, Ettlingcn, Germany). UV spectra were recorded on a PerkinElmer Lambda 35 spectrophotometer (PerkinElmer, Waltham, MA, USA). Semi-preparative HPLC was performed on an Agilent 1100 liquid chromatography (Agilent, Santa Clara, CA, USA) with an YMC (10 × 250 mm, 5 μm) column. GC analysis was performed with a capillary column (30 m × 0.32 mm × 0.5 μm) on an Agilent 7890A GC. Silica gel (200–300 mesh, Qingdao Marine Chemical Inc., Qingdao, China), ODS (50 μm, YMC, Kyoto, Japan), and Sephadex LH-20 (Pharmacia Biotech AB, Stockholm, Sweden) were used for column chromatography.

### 3.2. Plant Material

The barks of *C. cassia* were collected at Qujing, Yunan Province, China, in July 2010. The plant material was identified by Prof. Changgong Zhang at the School of Pharmacy, Tongji Medical College, Huazhong University of Science and Technology. A voucher specimen (No. 2010-0703) was deposited in the herbarium of Hubei Key Laboratory of Natural Medicinal Chemistry and Resource Evaluation, Tongji Medical College, Huazhong University of Science and Technology.

### 3.3. Extraction and Isolation

The air-dried stem barks of *C. cassia* (25 kg) were extracted with 95% ethanol at room temperature three times. The EtOH extract was concentrated *in vacuo* (2.6 kg), and then suspended in water and partitioned successively with petroleum ether, CHCl_3_, EtOAc, and *n*-BuOH. The EtOAc soluble extract was subjected to column chromatography (CC) (100–200 mesh) over silica gel, eluting with a gradient of CHCl_3_/MeOH to yield fractions 1–6. Fraction 3 (16 g) was chromatographed on YMC gel column (ODS) eluted with MeOH/H_2_O (10%–70%), and on silica gel with a gradient of CHCl_3_/MeOH (from 25:1 to 10:1) to afford compounds **2** (25 mg), **3** (18 mg), and **4** (20 mg). Compound **1** (15 mg) was isolated from fraction 4 and purified by semipreparative HPLC (MeOH/H_2_O, 30:70). Fraction 5 (21 g) was chromatographed on a silica gel CC with a gradient of CHCl_3_/MeOH (15:1) to yield two subfractions 5.1 and 5.2. Compound **5** (15 mg) was purified from fraction 5.1 by semipreparative HPLC (MeOH/H_2_O, 20:80), compounds **6** (8 mg), **7** (5 mg), and **8** (10 mg) were isolated from fraction 5.2.

### 3.4. Acid Hydrolysis

A solution of **1** (2.0 mg), in 2 M aqueous CF_3_COOH (2.0 mL) was heated at 60 °C for 2 h in a water bath [13]. The reaction mixture was diluted in H_2_O (2.0 mL) and extracted with EtOAc (2.0 mL × 3), then the aqueous layer was concentrated to remove CF_3_COOH. The residue was dissolved in pyridine (1.0 mL), to which L-cysteine methyl ester hydrochloride (2.0 mg) in pyridine (1.0 mL) was added. Then, the mixture was kept at 60 °C for 30 min. After the reaction mixture was dried *in vacuo*, the residue was trimethylsilylated with 1-trimethylsilylimidazole (0.4 mL) at 60 °C for 30 min in a water bath. Finally, the mixture was partitioned between *n*-hexane and H_2_O (1 mL each) and the *n*-hexane extract was analyzed by gas chromatography (GC) under the following conditions: column temperature, 250 °C; injection temperature, 250 °C; carrier N_2_ gas; flow rate 1.0 mL/min. In the acid hydrolysate of 1, d-glucose and d-apiose were confirmed by comparison of the retention times of their derivatives with those of d-glucose and d-apiose derivatives prepared in a similar way, which showed retention times of 10.03 and 15.62 min.

*Methyl 2-phenylpropanoate-2-O-β-d-apiofuranosyl-(1→6)-O-β-d-glucopyranoside* (**1**): amorphous powder; ${\left[\alpha \right]}_{D}^{25}$ −65.1 (*c* = 0.47, MeOH); UV (MeOH) λ_max_ (log *ε*) = 213 (2.9) nm; IR (KBr) ν_max_ 3391, 2926, 2884, 1719, 1493, 1236, 1067, and 756 cm^−^^1^; ^1^H-NMR (CD_3_OD, 400 MHz) and ^13^C-NMR (CD_3_OD, 100 MHz) see Table 1; HRESIMS *m/z*: 497.1616 [M+Na]^+^ (calcd for C_21_H_30_O_12_Na, 497.1629). 

### 3.5. Lymphocyte Proliferation Test

Splenic lymphocytes were prepared as previously described by Kawaguchi *et al*. [14]. The lymphocyte proliferation was determined by WST-8 assay using Cell Counting Kit-8 (Dojindo) [15]. The prepared spleen cells (2.5 × 10^6^ cells) were seeded into each well of a 96-well microplate, and various concentrations of compounds and 5 μg/mL of concanavalin A (Con A, from *Canavaliaensiformis* Type III), for selective stimuli on T cells were added, cyclosporine A (CsA) was used as a positive control. After being cultured at 37 °C with 5% CO_2_ for 48 h, 20 μL WST-8 was added to each well. The absorbance at 450 nm with a 600 nm reference was detected on a microtiter plate reader (Bio-Rad 680). All optical density (OD) values shown are the mean of triplicate sample ± SD. Controls with and without ConA and LPS were used to establish the baseline proliferation for stimulated and unstimulated cells. The lymphocyte proliferation rate was evaluated according to our reported procedure [5].

## 4. Conclusions

A new phenolic glycoside, methyl 2-phenylpropanoate-2-*O*-*β*-d-apiofuranosyl-(1→6)-*O*-*β*-d-glucopyranoside (**1**), was isolated from the barks of *C. cassia*, together with seven known compounds (**2**–**8**). The structure and relative configuration of the new isolate was elucidated by careful interpretation of spectroscopic data. This is the first report of samwiside (**5**) in the genus of *Cinnamomum*. Compounds **1**, **2** and **5**–**8** were tested for their *in vitro* immunomodulatory activities. Compounds **2** and **6** showed weak inhibition against ConA-induced T cell proliferation at the dosage of 12.5–200 μM, and the inhibitory effects are not dose-dependent. Interestingly, at a concentration of 200 μM, compound **8** significantly inhibited ConA-induced T cell proliferation with an inhibition ratio of 80.1%, whilst at low concentrations of 25 and 12.5 μM, stimulated the proliferation of T cell.
